# Small molecule Mcl-1 inhibitor for triple negative breast cancer therapy

**DOI:** 10.3389/fcell.2024.1408107

**Published:** 2024-09-20

**Authors:** Shengli Dong, Suresh K. Alahari

**Affiliations:** ^1^ TYK Medicines, Inc., Zhejiang, China; ^2^ Department of Biochemistry and Molecular Biology, LSHSC School of Medicine, New Orleans, LA, United States; ^3^ Stanley S. Scott Cancer Center, LSUHSC School of Medicine, New Orleans, LA, United States

**Keywords:** cancer, apoptosis, inhibitor, drug, therapy

## Abstract

Apoptosis is an evolutionarily conserved cell death pathway that plays a crucial role in maintaining tissue homeostasis, orchestrating organismal development, and eliminating damaged cells. Dysregulation of apoptosis can contribute to the pathogenesis of malignant tumors and neurodegenerative diseases. Anticancer drugs typically possess the capacity to induce apoptosis in tumor cells. The Bcl-2 protein family, consisting of 27 members in humans, serves as the key regulator of mitochondrial function. This family can be divided into two functional groups: anti-apoptotic proteins (e.g., Bcl-2, Bcl-xl, Mcl-1) and pro-apoptotic proteins (e.g., Bad, Bax). Mcl-1 exerts its function by binding pro-apoptotic Bcl-2 proteins thereby preventing apoptosis induction. Overexpression of Mcl-1 not only correlates closely with tumorigenesis but also associates significantly with resistance towards targeted therapy and conventional chemotherapy. Effective induction of apoptosis can be achieved through inhibition or interference with Mcl-1. Thus, this mini review discusses existing Mcl-1 inhibitors.

## Introduction

Breast cancer has surpassed lung cancer to become the most common cancer in the world in 2021 (Siegel et al., 2021). Triple negative breast cancer (TNBC) is generally defined as a type of breast cancer in which estrogen receptor (ER), progesterone receptor (PR) and human epidermal growth factor receptor 2 (HER2) are all expressed negatively, accounting for about 12%–17% of all breast cancers (Foulkes et al., 2010). TNBC is a very heterogeneous type of breast cancer. According to gene expression profiling, TNBC can be divided into four distinct subtypes: immunomodulatory type (IM), luminal androgen receptor type (LAR), basal-like immunosuppression type (BLIS), and interstitial type (MES) (Jiang et al., 2019). TNBC is relatively aggressive in clinics, with an earlier age of onset, more visceral metastases, and faster distant recurrence compared to patients with other breast cancer subtypes, which also makes the treatment of TNBC more difficult and urgent. Progress in the treatment of TNBC has lagged behind other subtypes over the past few decades, and there are currently no specific treatment guidelines for TNBC. TNBC treatment is generally performed according to the standard breast cancer treatment with a median survival of only 13 months. Tumor therapy is becoming more precise and personalized. However, there is a shortage of effective targeting therapeutic for TNBC at present.

**FIGURE 1:**
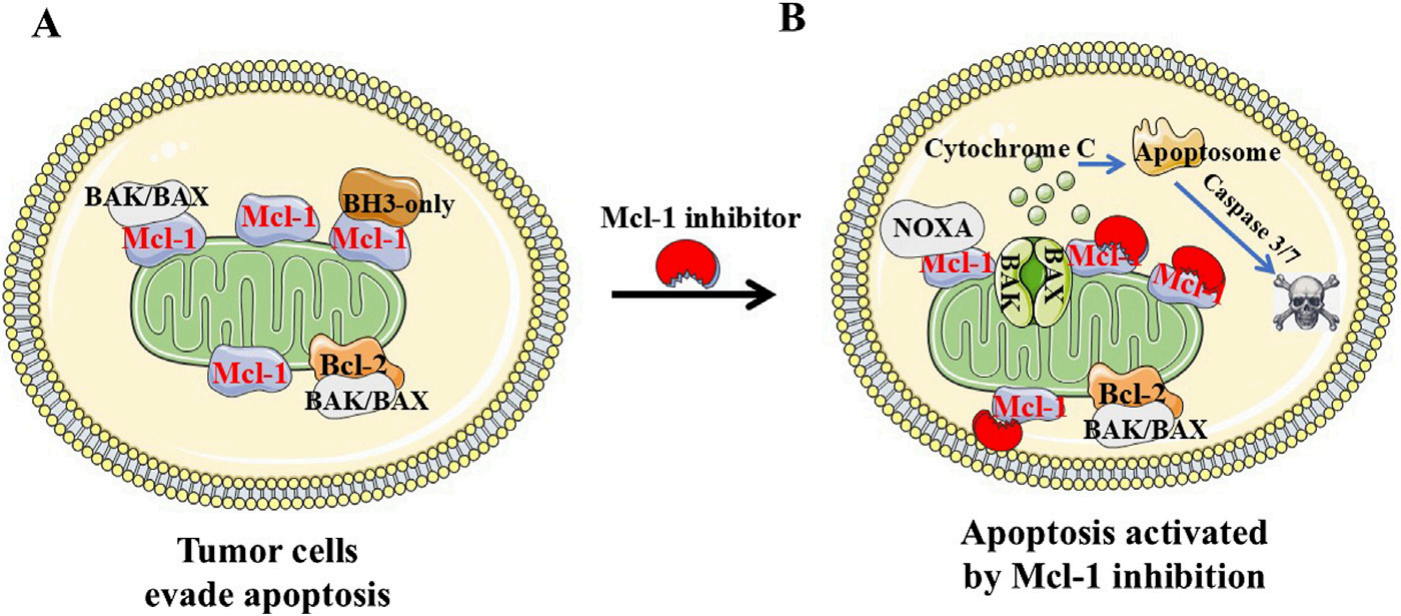
Diagram of pro-apoptosis and anti-apoptosis regulation in tumor cells. **(A **and **B)** Pro-apoptotic protein Bax and Bak are “effectors.” Bak/Bax oligomerizes by binding to “activators” (Bim, Bid, and Puma) at the outer mitochondrial membrane, resulting in cytochrome-c release, apoptosome formation and caspase-dependent apoptosis. Anti-apoptotic Bcl-2 family proteins (Bcl-2, Bcl-A1, Bcl-xL, Bcl-w, and Mcl-1) favor cell survival by binding and sequestering effectors and activators. Cancer cells evade apoptosis by increasing levels of anti-apoptotic Bcl-2 proteins including Mcl-1. Mcl-1 inhibitor such as NSC-260594 could inhibit Mcl-1 and induce cancer cell apoptosis in TNBCs.

Apoptosis is an evolutionarily conserved cell death pathway that plays a crucial role in maintaining tissue homeostasis, orchestrating organismal development, and eliminating damaged cells. Dysregulation of apoptosis can contribute to the pathogenesis of malignant tumors and neurodegenerative diseases. Anticancer drugs typically possess the capacity to induce apoptosis in tumor cells. The Bcl-2 protein family, consisting of 27 members in humans, serves as the key regulator of mitochondrial function. According to the number or the function of Bcl-2 homologous (BH) motifs, the Bcl-2 family can be classified into three subgroups: pro-survival/anti-apoptotic proteins, which have multiple BH motifs (BH1−BH4) including Bcl-2, Bcl-xL, Mcl-1, Bcl-W and BFL1; apoptotic effector proteins Bak, Bax and Bok containing multiple (BH1−BH4) motifs; and BH3-only proteins, which bear single BH3 motif and play essential role in the apoptosis pathway as activator (Bim, Bid, Puma) or sensitizer (Bad, Noxa, Bik, Bmf) proteins (Ashkenazi et al., 2017). Under cellular homeostasis, Mcl-1 binds and sequesters both BH3-only activator as well as effector molecules. In the presence of an apoptotic signal such as radiation or chemotherapy, free BH3-only proteins directly bind to Mcl-1 with high affinity, initiate the disassociation of sequestered Bak and Bax. Furthermore, activated Bak/Bax are capable to form homo-oligomerization, and insert into mitochondrial outer membrane resulting in cytochrome c releasing and caspase activation. A delicate balance between anti-apoptotic and pro-apoptotic proteins must be maintained for the regulation of cell survival (Figure 1). Apoptosis is predominantly achieved through two pathways, namely, the extrinsic receptor pathway and the intrinsic mitochondrial pathway. In the extrinsic receptor pathway, cytokine ligands such as TNF, FAS, and TRAIL bind to death receptors forming large DISC complexes that subsequently recruit and activate Caspase 8 and 10, ultimately activating downstream caspase 3/7 to accomplish apoptosis. Due to challenges associated with targeting the extracellular receptor pathway (e.g., FAS-induced severe hepatotoxicity) and a lack of reliable screening markers for TRAIL targeting, mitochondria-dependent apoptosis serves as one of the primary mechanisms employed by over 90% of anticancer drugs for cancer cell eradication. In the context of tumor therapeutics, particular attention is given to the anti-apoptotic Bcl-2 proteins including Bcl-2 and Mcl-1 that are frequently overexpressed in tumors. Such overexpression confers resistance to chemotherapy, radiotherapy, and targeted therapies (Holohan et al., 2013). Small-molecules targeting pro‐apoptotic Bcl-2 family proteins are very promising in cancer treatment. ABT-737 was the first BH3 mimetic molecule with nanomolar affinity for Bcl-xL and Bcl-2. However, ABT-737 exhibited low oral bioavailability because of its poor solubility, permeability, and metabolic properties (Deng et al., 2007). To improve its oral bioavailability and cellular efficacy, researchers further optimized ABT-737 and developed an orally active molecule ABT-263 (navitoclax). ABT-263 binds with high affinity to Bcl-2, Bcl-XL, and Bcl-W, but not to Mcl-1. However, ABT-263 induced rapid and dose-dependent platelet toxicity in clinics, possibly a consequence of the potent Bcl-xL inhibition (Tse et al., 2008). This prompted the development of a selective Bcl-2 inhibitor without Bcl-xL inhibition. Researchers successfully developed ABT-199 (Venetoclax) with super Bcl-2 selectivity that exhibited efficacy in hematologic tumor but spared platelets (Souers et al., 2013). Venetoclax received FDA approval in 2016 for the treatment of relapsed and refractory chronic lymphocytic leukemia (CLL) with 17p deletion and small lymphocytic lymphoma (SLL) as monotherapy, and acute myeloid leukemia (AML) in combination with decitabine, azacitidine, or cytarabine. The FDA approval of venetoclax is a milestone of cancer therapy by targeting anti-apoptotic proteins. However, the limitations and deficiencies of venetoclax monotherapy were gradually exposed with the extensive clinical investigations, which mainly include limited monotherapy activity; complex drug resistance mechanisms; and on-target dose limiting toxicity. Therefore, there are the urgent unmet medical needs in targeting Bcl-2 anti-apoptotic proteins including how to broaden the anti-tumor potential; circumvent the on-target toxicity; and overcome the drug resistance. Accumulated evidence suggested that the efficacy of ABT-737, ABT-263, and Venetoclax was reduced because tumor cells upregulated the compensatory Mcl-1. Tumor cells regained sensitivity to ABT-737 or Venetoclax as Mcl-1 gene was knocked out in leukemia cells, which suggested Mcl-1 was the primary reason of resistance to anti-Bcl-2 compounds (Nguyen et al., 2007; Punnoose et al., 2016). In addition, while solid tumors depend more on Mcl-1, hematological malignancies are more dependent on both Mcl-1 and Bcl-2. Mcl-1 knockout significantly suppressed the growth of both solid and blood cancers, the latter were more affected. Bcl-2 inhibition only affected the survival of hematological tumor cells, but not on solid tumor cells (Bolomsky et al., 2020). Furthermore, Mcl-1 has a more electropositive binding surface resulting in strong binding affinities for Bak, Bim, Noxa and Puma, and weak binding affinity for Bmf and Bad. Together, targeting Mcl-1 has advantages over targeting other anti-apoptotic proteins.

Mcl-1 exerts its function by binding pro-apoptotic Bcl-2 proteins thereby preventing apoptosis induction. Overexpression of Mcl-1 not only correlates closely with tumorigenesis but also associates significantly with resistance towards targeted therapy and conventional chemotherapy. Effective induction of apoptosis can be achieved through inhibition or interference with Mcl-1 (Goodwin et al., 2015). Similar to other Bcl-2 family members, Mcl-1 contains a transmembrane domain at its C-terminal region which facilitates its localization on various cellular membranes including the outer mitochondrial membrane. Localized within mitochondria, and playing a pivotal role in regulating mitochondrial apoptotic pathways, Mcl-1 is becoming an attractive target for cancer treatment. Mcl-1 protein has eight alpha helices (α1−α8). α2−α5 helices form a BH3-binding hydrophobic groove which has four hydrophobic pockets (P1−P4) and a conserved aspartic acid at amino acid 263 (Asp263), where the helical BH3 region of other Bcl-2 family members binds. The results of mutagenesis studies revealed that amino acid residues at positions i, i+4, i+7, and i+11 within the Bim establish nonpolar interactions with P1 to P4 pockets in Mcl-1 binding groove. The aspartic acid at i+9 in Bim form a strong salt bridge interaction with Asp263 in Mcl-1. Therefore, interfering the protein−protein interactions (PPIs) between Mcl-1 and BH3-only proteins Bim, Bak, and Bax has become an effective therapeutic strategy. However, targeting PPIs associated with Mcl-1 using inhibitors faces challenges due to its flat surface binding groove and discontinuous hot-spot areas (Holohan et al., 2013; Deng et al., 2007). Currently there are 17 ongoing global developments on Mcl-1 inhibitors and 7 molecules in clinical stage (Table 1) (Negi and Murphy, 2021; Wang et al., 2021).

**TABLE 1:** The information of Mcl-1 inhibitors in clinical trials.

MCL-1 inhibitor	Company	Study	Conditions	Status	No. Enrolled	Stage	NCT No.
PRT1419	Prelude Therapeutics	PRT1419 as Monotherapy or in Combination With Azacitidine or Venetoclax in R/R Myeloid or B-cell Malignancies	AML, CLL	Active, not recruiting	132	Phase I	NCT05107856
		A Study of PRT1419 in Patients With Advanced Solid Tumors	Relapsed or refractory solid tumors, including breast, lung, sarcoma and melanoma et al	Completed	26	Phase I	NCT04837677
		A Study of PRT1419 in Patients With Relapsed/Refractory Hematologic Malignancies	MM, NHL, AML, CMML, MDS or MPN	Completed	16	Phase I	NCT04543305
AZD5991	AstraZeneca	Study of AZD5991 Alone or in Combination With Venetoclax in Relapsed or Refractory Haematologic Malignancies	NHL, CLL, TCL, MM,AML MDS	Terminated	70	Phase I/II	NCT03218683
AMG-176	Amgen and AbbVie	A Study of Venetoclax and AMG 176 in Patients With Relapsed/Refractory Hematologic Malignancies	AML,NHL, DLBCL	Terminated	9	Phase I	NCT03797261
	Amgen	AMG 176 With Azacitidine in Subjects With Myelodysplastic Syndrome/Chronic Myelomonocytic Leukemia	MDS, CML	Completed	9	Phase I	NCT05209152
		AMG 176 First in Human Trial in Participants With Relapsed or Refractory Multiple Myeloma and Participants With Relapsed or Refractory Acute Myeloid Leukemia	MM, AML	Active, not recruiting	142	Phase I	NCT02675452
AMG-397	Amgen	Safety, Tolerability, Pharmacokinetics and Efficacy of AMG 397 in Subjects With Selected Relapsed or Refractory Hematological Malignancies	MM, AML, MDS, NHL	Terminated	24	Phase I	NCT03465540
ABBV-467	AbbVie	A Study of the Safety and Tolerability of ABBV-467 in Adult Participants With Relapsed/Refractory (R/R) Multiple Myeloma	MM	Terminated	8	Phase I	NCT04178902
MIK665/S64315	Novartis	Phase I Study of MIK665, a Mcl-1 Inhibitor, in Patients With Refractory or Relapsed Lymphoma or Multiple Myeloma	MM, Lymphoma, DLBCL	Completed	31	Phase I	NCT02992483
		Phase I/II Trial of S64315 Plus Azacitidine in Acute Myeloid Leukaemia	AML	Active, not recruiting	17	Phase I/II	NCT04629443
		Phase I Study of S64315 Administred Intravenously in Patients With Acute Myeloid Leukaemia or Myelodysplastic Syndrome	AML, MDS	Completed	38	Phase I	NCT02979366
		Phase I Dose Escalation Study of Intravenously Administered S64315 in Combination With Orally Administered Venetoclax in Patients With Acute Myeloid Leukaemia	AML	Completed	36	Phase I	NCT03672695
GS-9716	Gilead Sciences	Study to Evaluate the Safety, Tolerability, and Pharmacokinetics of GS-9716 as Monotherapy and in Combination With Anticancer Therapies in Adults With Solid Malignancies	Solid Malignancies	Recruting	195	Phase I	NCT05006794

Note: AML, acute myeloid leukemia; CMML, chronic myelomonocytic leukemia; CLL, chronic lymphocytic leukemia; DLBCL, diffuse large B-cell lymphoma; MDS, myelodysplastic syndromes; MM, multiple myeloma; NHL, non-Hodgkin lymphoma; R/R, refractory or relapsing; TCL, T-cell lymphoma.

In a recent published paper Dong et al. (2023) found that NSC260594 treatment efficiently killed TNBC cells, but not the mouse embryonic fibroblast (MEF) cells, in a dose- and time-dependent manner. NSC260594 came from the NCI diversity set IV compound library. Little is known about the function of NSC260594. A few previous studies indicated that NSC260594 might have antiviral and anti-parasitic activity. Beekman et al. (2016) found that NSC146771 could displace a 19-residue NoxaB peptide (AAQLRRIGDKVNLRQKLLN) from Mcl-1 binding site. NSC260594 methylates nitrogen on quinolinium of its analog NSC146771 and shares a highly similar stereo structure with NSC146771. Therefore, Dong et al. (2023) hypothesize that NSC260594 might inhibit Mcl-1. Indeed, NSC260594 treatment completely inhibited Mcl-1 expression in both MDA-MB 231 and its more aggressive derivative 4175 cells. The data also suggested that NSC260594 suppressed Mcl-1 expression through downregulation of Wnt signaling pathways in TNBCs. Mcl-1 plays a key role in drug resistance. The deregulation of Wnt signaling pathway associates with breast cancer stem cells (CSCs) (Lamb et al., 2013). Dong et al. (2023) hypothesized that NSC260594 treatment could overcome drug resistance not only through Mcl-1 inhibition but also through CSCs suppression. As expected, inhibition of anti-apoptotic protein Mcl-1 by NSC260594 promoted apoptosis. NSC260594 treatment also not only significantly decreased the expression of cancer stem cell maker ALDH1/2, but also decreased the population of CD44^high^ and CD24^low^ cells by flow cytometry analysis. As Mcl-1 overexpression often results in resistance to target therapies, the combination of targeted agents and Mcl-1 inhibitors could be an option to overcome this resistance. PI3K/AKT/mTORC1 aberrant activation is one of the most commonly deregulated pathways in TNBCs (Cancer Genome Atlas, 2012), and Mcl-1 overexpression is one reason of drug resistance to everolimus therapy in TNBCs (Mills et al., 2008). The combination of NSC260594 and everolimus actually performed synergistically to kill human TNBC cells and human 3D TNBC organoids.

Despite notable progress made thus far, small molecule inhibitors targeting Mcl-1 still face certain challenges. Most of the reported Mcl-1 inhibitors have poor anti-tumor activity due to poor physical and chemical properties, low mitochondrial membrane penetration, and high serum protein binding rate (Negi and Murphy, 2021; Wang et al., 2021). Since the IC_50_ of NSC260594 falls in a range between 0.5 and 10 μM in TNBC cells, the molecule is a good lead compound for further optimization. NSC260594 is very difficult to dissolve in aqueous phase. There has been a high priority to improve DMPK properties through increasing the solubility. In addition, the computational docking of the NSC260594 with Mcl-1 found that linear NSC260594 loosely fit in the BH3 binding groove of Mcl-1 (PDB ID: 6U6F, data not shown). The loose interaction NSC260594 with Mcl-1 is the probably reason why many proteins were downregulated by the NSC260594. In a recently published JMC paper, Kump et al. (2020) found that U-shaped molecules increased the binding affinity with Ki values of 100 nM. It might optimize and make NSC260594 more selective to Mcl-1 with the same strategy.

**FIGURE 2:**
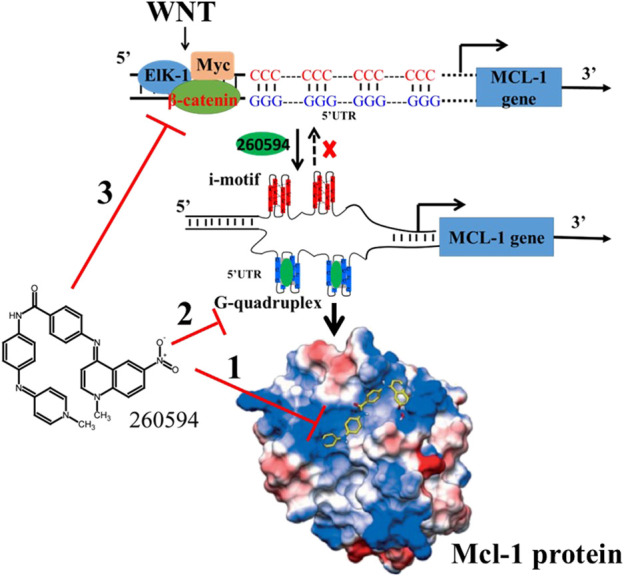
NSC260594 can regulate Mcl-1 expression at multiple levels: 1) via direct binding to Mcl-1 groove pocket; The crystal structure is adopted from Beekman et al. (2016). 2) by stabilizing G-quadruplexes in Mcl-1 promoter region; and 3) by suppressing Wnt signaling pathway.

Mcl-1 is a short-lived protein. Once Mcl-1 gene transcription or Mcl-1 mRNA translation is interrupted, the existing pool of Mcl-1 will be quickly degraded by the proteasome. Mcl-1 can be either directly or indirectly regulated at multiple levels involving transcriptional, post-transcriptional and post-translational processes (Negi and Murphy, 2021). As illustrated in Figure 2, NSC260594 could bind the shallow BH3 hydrophobic groove in the Mcl-1 protein surface. NSC260594 can regulate Mcl-1 through modulating Wnt signaling pathway. In addition, a DNA secondary structure named G-quadruplex is an emerging therapeutic target in oncology (Kosiol et al., 2021). There is a G/C rich area in the promoter region of human Mcl-1 gene (Bingle et al., 2000). This G/C rich region might form a similar G-quadruplex in Bcl-2 promoter region. NSC260594 has the potential to suppress Mcl-1 expression through stabilizing G-quadruplexes. Therefore, it is important to provide more solid evidence whether the G/C rich area can form G-quadruplexes in the promoter region or at the splice sites of human Mcl-1 gene, and whether NSC260594 can directly regulate Mcl-1 gene expression through interaction with G-quadruplexes.

Although there has been a lot of efforts in recent years to improve the affinity of Mcl-1 inhibitors through structural optimization, due to the neglect of the distribution of Mcl-1 inhibitors in cells, off-target effects have been exhibited in cells. Cardiomyocytes are the highly specialized cells that make up the cardiac muscle. Cardiomyocytes pump blood to circulate the human body through a contraction-relaxation cycle. If cardiomyocytes lose function, the muscle won't pump blood as well as it should be, and heart failure occurs. Using Mcl-1 conditional knocked-out mice, Opferman’s group found that cardiac-specific ablation of Mcl-1 in cardiomyocytes resulted in a rapidly fatal dilated cardiomyopathy, which suggested that pharmacological Mcl-1 inhibition might result in an unexpected cardiotoxicity (Wang et al., 2013). In addition, Thomas and Gustafsson (2013) found that deletion of Mcl-1 in mouse myocardium would be incapable of activating autophagy in the heart, which suggested that Mcl-1 had critical functions to facilitate autophagy and mitophagy in myocytes other than inhibiting apoptosis. As we previously described, multiple Mcl-1 inhibitors have been evaluated in clinical trials. Many of these inhibitors bind to Mcl-1 very potent with a subnanomolar affinity (Table 2). It was thought that Mcl-1 blockade might result in the potential cardiotoxicity in patients. Indeed, a few trials were terminated because of cardiac toxicity (AMG-176, NCT02675452 and NCT03797261) (Wang et al., 2021; Tantawy et al., 2023), or for potential safety reasons (AZD-5991, NCT03218683). A switch in apoptosis dependency from Bcl-2 to Mcl-1 results in venetoclax resistance in lymphoid malignancies. However, Mcl-1 inhibitor AZD5991 combination with venetoclax led to an increase in cardiac “laboratory parameters” in one patient, and AstraZeneca voluntarily suspended the drug development (Ong et al., 2022; Zhang et al., 2020). Therefore, it is urgent to improve the targeted binding efficiency of NSC 260594 to mitochondria and reduce the potential off-target effects, thereby enhancing the anti-tumor activity of Mcl-1 inhibitors and reducing toxicity. Human ether-a-go-go-related gene (hERG) is a potassium channel that has an essential role in the repolarization of cardiac action. Decreases in hERG activity indicate the potential risk of fatal cardiac arrhythmias. Therefore, it is a good solution to evaluate the hERG activity of NSC 260594 carefully to avoid the drug potential cardiotoxicity.

**TABLE 2:** The summary of the current potent inhibitors, their targets and their *in vitro* and *in vivo* efficacies.

Compound	Inventor	Target	Affinity (Mcl-1)	*In vitro* (EC_50_, nM)				*In vivo* efficacy	References
				AMO-1	H929		DLD-1		
ABBV-467	Abbvie	Mcl-1	Ki < 0.01 nM	0.16	0.47	3.91	>10,000	TGI ranging from 46%–97% following doses of 3.13, 6.25, or 12.5 mg/kg in AMO-1 CDX model	Deng et al. (2024)
AMG-176	Amgen	Mcl-1	Ki = 0.06 nM	90.6	195	106	>10,000	AMG 176 is efficacious in OPM-2 xenograft model, The combination of AMG 176 and venetoclax is synergistic in MOLM13 orthotopic tumor xenografts	Caenepeel et al. (2018)
AMG-397	Amgen	Mcl-1	Ki = 0.015 nM	13.5	13	43.6	>10,000	Once or twice weekly dose at 50 mg/kg resulted in 9 of 10 mice tumor free at the end of the study in OPM2 xenografts	Caenepeel et al. (2020)
AZD5991	AstraZeneca	Mcl-1	Ki = 0.2 nM	22.9	31.7	34.9	>10,000	A single i.v. dose of 100 mg/kg induced complete tumor rgression in MV4-11 tumor model 7 days after treatment	Tron et al. (2018)
S64315/MIK665	Servier/Novartis	Mcl-1	Ki = 0.026 nM	2.06	4.75	10.87	4,750	A dose-dependent antitumor activity was observed with TGImax of 89.1%, 115.8%, and 162.8% at 3.125 mg/kg, 6.25 mg/kg, and 12.5 mg/kg, respectively in AMO1 xenografted mice by i.v. for 5 consecutive days	Szlavik et al. (2020)
GS-9716	Gilead Sciences	Mcl-1	N/A	Median GI_50_ = 470 nM for breast cancer cells; Median GI_50_ = 30 nM for a panel of hematological cells				The combination of paclitaxel with GS- 9,716 completely inhibited tumor growth in TNBC HCC1187 CDX.	Matson et al. (2022)
JNJ-4355	Janssen Pharmaceutica	Mcl-1	K_i_ = 0.015 nM	MOLP8 (multiple myeloma) AC_50_ = 12 nM				A single i.v. dose resulted in complete tumor regression in MOLP8 CDX model	Rombouts et al. (2022)
PRT1419	Prelude Therapeutics	Mcl-1-Bim binding	IC_50_ = 6.6 nM	IC_50_ = 80 nM for OPM2 cell (24 h)				PRT1419 treatment shows rapid apoptosis induction and potent anti-tumor activity in multiple CDX and PDX mouse models	Bhagwat et al. (2021)

Efficacy of Mcl-1 inhibitors generally required high dose, which might result in undesired side effects due to off-target binding. Consequently, new drug classes such as proteolysis targeting chimera (PROTAC) have been developed since its concept was reported at beginning of this century. PROTAC consists of a ligand binding an E3 ligase conjugated to a small-molecule warhead binding the target protein through a linker. PROTAC mediates the formation of a ternary complex between an E3 ligase and target protein of interest (POI), and promotes the ubiquitination and subsequent degradation of the POI. Up to date, more than two thousand PROTACs have been developed to target 124 different POIs (Bekes et al., 2022). PROTACs have succeeded in degrading hormone receptors in clinical studies. For example, Arvinas’ androgen receptor PROTAC (ARV-766) has been well tolerated to date in phase III clinical study and is showing promising efficacy in the prostate cancer treatment (NCT05067140). Vepdegestrant (ARV-471) is a first-in-class estrogen receptor-degrading PROTAC in phase III clinical studies. Overall response rate (41.9%), median progression-free survival (11.2 months), and safety profile of vepdegestrant in combination with palbociclib were achieved in heavily treated patients in phase Ib study (NCT05654623). It received FDA fast track designation for the treatment of patients with ER+/HER2-metastatic breast cancer in February, 2024. Zhang group recently reported the selective Mcl-1 degrader C3 was much more active against the Mcl-1-driven H23 cells than the most potent selective Mcl-1 inhibitor A-1210477 (Wang et al., 2019). In addition, Derksen group developed Mcl-1 degrader dMCL1-2 through conjugating A-1210477 molecule to CRBN E3 ligase. dMCL1-2 degraded MCL1 at nanomolar concentrations in multiple myeloma (MM) OPM2 wild type cells (Papatzimas et al., 2019).

Chemotherapy regiments are currently the cornerstone of systemic therapy for TNBC. Microtubules have fundamental cellular functions and are targets of antitubulin chemotherapy. Microtubule-targeted agents such as paclitaxel are prescribed widely for TNBC. For example, the combination of platinum with paclitaxel/anthracycline/bevacizumab is a recommendation for more optimal neoadjuvant therapy in TNBC. Moreover, results from the one study demonstrated that atezolizumab in combination with nab-paclitaxel in the first-line treatment of advanced TNBC resulted in PFS benefits. The results of this study led to the first time FDA approval of atezolizumab for the first-line treatment of advanced TNBC (Narayan et al.). Unfortunately, paclitaxel often leads to the acquisition of drug resistance in TNBC, which is one of the main reasons for the tumor relapse and metastasis. Wertz et al. (2011) found that Mcl-1 is a key regulator of apoptosis triggered by antitubulin agent paclitaxel (Taxol). Phosphorylated Mcl-1 directs its interaction with FBW7, which is the E3 ligase substrate. Ubiquitylated Mcl-1 then directs it for proteasomal degradation (Thomas and Gustafsson, 2013). The results suggested that combination Mcl-1 inhibition and paclitaxel might restore sensitivity to antitubulin chemotherapeutics in TNBC. Precision medicines have progressed rapidly in TNBC treatment over the last decade. Olaparib monotherapy showed significantly superior to placebo in early-stage TNBC patients with gBRCA mutations. The FDA granted accelerated approval to Trodelvy^®^ (sacituzumab govitecan-hziy), which is the world’s first ADC for the TNBC treatment (Fleming et al., 2021). In current manuscript, we focus on Mcl-1 targeting therapy for the TNBC. In fact, Mcl-1 inhibitor can induce apoptosis by monotherapy or combination treatments with other therapies, which allows various cancers to obtain benefit from it. For example, most multiple myeloma (MM) cells are dependent on Mcl-1 for survival, the relapsed MM patients are often with higher Mcl-1 protein expression. Mcl-1 targeting therapy is an effective strategy for MM treatment. Mcl-1 protein was found overexpressed in 51% specimens of 149 hepatocellular carcinoma (HCC) patients. Mcl-1 knocked down led to significant therapeutical activity against HCC cells *in vitro* (Sieghart et al., 2006). Mcl-1 is frequently upregulated in acute myeloid leukemia (AML) cells, particularly at time of relapse. S63845 is a selective Mcl1-inhibitor and exhibit high potency in myeloma, AML, and lymphoma. The combination treatment of S63845 and the MEK1/2-inhibitor trametinib was synergistic in AML cells and primary patient’s samples (Seipel et al., 2019).

Drug resistance inevitably occurs after a period of targeted therapy for tumors. Mitogen-activated protein kinases (MAPKs) regulate a complex intracellular signaling network by different cell surface receptors. Each MAPK cascade consists of a MAPK, a MAPK kinase (MAP2K), and a MAP2K kinase (MAP3K). The terminal MAPKs are the p38, Erk, JNK kinases. The mutations of human RAS genes, including HRAS, NRAS, and KRAS, occur in approximately 20%–30% of cancers. RAF is recruited to the cell membrane upon RAS activation, which triggers sequential activation of the MEK1/MEK2 and ERK. Activated ERKs translocate into the nucleus where they mediate multiple genes required for survival and proliferation. RAS-RAF–MEK–ERK cascade has an essential role in the regulation of cell proliferation and survival. Aberrant constitutive activation of this pathway frequently happens in human cancers. For example, activating mutations in B-RAF (BRAF) have been identified in approximately 50% of melanoma. V600E and V600K account for 95% of the BRAF mutations (Davies et al., 2002). B-RAF inhibitors vemurafenib and dabrafenib, and MEK1/2 inhibitor trametinib have improved the clinical outcomes of melanoma patients with BRAF V600E/K mutant. However, acquired resistance and disease relapse typically occur through ERK1/2 reactivation (Cohen et al., 2021). ERK1/2 signalling increases the expression of anti-apoptotic Mcl-1, Bcl-2 and Bcl-XL and inhibits the pro-apoptotic BH3-only proteins such as Bim, Puma and Bad. Sale et al. found that Bcl-XL protein was strikingly lower in melanoma than CRC, NSCLC and pancreatic in a panel 64 cancer cell lines. The Mcl-1:Bcl-XL protein ratio was five times higher in melanoma cell lines and patient samples. Combination treatment of BRAFi or MEKi with Mcl-1i can selectively kill melanoma cells. BRAF or MEK1/2 inhibitors are synergistic with the Mcl-1 inhibitor AZD5991 *in vitro* and *in vivo*. AZD5991 enhances the efficacy of an ERK inhibitor in mouse CDX models (Sale et al., 2019). Montero et al. (2019) also found that Mcl-1 is a key driver of adaptive survival in tumors that treated with targeting therapies. They demonstrated that combination inhibition of Mcl-1 and RAF-MEK-ERK pathway yielded dramatic therapeutic activity (Sale et al., 2019; Montero et al., 2019) Oakes group found that Mcl-1 drives breast cancer invasion, and suggested that Mcl-1 inhibition could be used in combination with medicine targeting Src kinases to suppress TNBC metastasis (Young et al., 2016).

Understanding which cells may be susceptible to Mcl-1 inhibition would help give more efficient therapy. To identify predictive biomarkers of response to AM-8621, an analog of AMG 176, Caenepeel et al. assessed the relationship between sensitivity and genetic features using a 952-cancer cells panel. They found that expression or copy-number variation of 165 genes was associated with drug response across the whole panel cell lines. The strongest predictor of AM-8621 resistance was high Bcl-xL expression, while the strongest predictor of sensitivity was high Bak expression (Caenepeel et al., 2018). Ramsey et al. (2018) found that BH3 profiling could predict response to Mcl-1 Inhibition. Resistance to inhibition of Mcl-1 was negatively correlated with the sensitivity to Bcl-2 inhibition. Combination treatment of Mcl-1 inhibitor VU661013 and Bcl-2 inhibitor Venetoclax was synergistic in PDX Models (Ramsey et al., 2018). Campbell et al. (2018) found that high Mcl-1 expression was associated with poor outcome in TNBC. The data supported the therapeutic targeting of Mcl-1 in TNBC (Campbell et al., 2018). These studies provided a rationale for combining targeting agents with Mcl-1 inhibitors for TNBC in the clinic. Multiple Mcl-1 inhibitors are examined in clinical trials. Multiomics data from these clinical trials may provide more clues to biomarkers of drug response.

Cell lines have been used to identify the mechanisms of resistance to Mcl-1 inhibition. The resistant cells showed overexpression of Bcl-2 or BCL-xL; c-Myc overexpression; loss of TP53 or Bax; drug efflux through MDR1; and Mcl-1 L267V mutation. In addition, tumor stromal microenvironment can also provide resistance to the action of Mcl-1 inhibition (Tantawy et al., 2023). Overall, the more studies are warranted to fully understand the potential and limitations of Mcl-1 inhibitors in cancer therapy. With the deepening of the understanding of drug resistance mechanisms and predictive biomarkers, it is expected that more improvements such as Mcl-1 inhibitor in the therapeutic effect of TNBC, coupled with the gradual update and implementation of clinical diagnosis and treatment guidelines, will bring survival benefits to more patients and conquer the refractory TNBC.
